# Single Nucleotide Polymorphisms of the *ERAP1* Gene and Risk of NSCLC: A Comparison of Genetically Distant Populations, Chinese and Caucasian

**DOI:** 10.1007/s00005-016-0436-4

**Published:** 2017-01-12

**Authors:** Yufeng Yao, Andrzej Wiśniewski, Qiangli Ma, Aneta Kowal, Irena Porębska, Konrad Pawełczyk, Jiankun Yu, Joanna Dubis, Natalia Żuk, Yingfu Li, Li Shi, Piotr Kuśnierczyk

**Affiliations:** 1 Yunnan Key Laboratory of Vaccine Research and Development on Severe Infectious Disease, Institute of Medical Biology, Chinese Academy of Medical Sciences (CAMS) and Peking Union Medical College (PUMC), Kunming, Yunnan China; 2 Laboratory of Immunogenetics and Tissue Immunology, Hirszfeld Institute of Immunology and Experimental Therapy, Polish Academy of Sciences, Wrocław, Poland; 3 Department of Thoracic Surgery, The No. 3 Affiliated Hospital of Kunming Medical University, Kunming, China; 4 Department and Clinic of Pulmonology and Lung Cancer, Wrocław Medical University, Wrocław, Poland; 5 Department and Clinic of Thoracic Surgery, Wrocław Medical University, Wrocław, Poland; 6 Research and Development Centre, Regional Specialist Hospital in Wrocław, Wrocław, Poland; 7 Department of Geriatrics, The No. 1 Affiliated Hospital of Kunming Medical University, Kunming, 650032 Yunnan China

**Keywords:** Non-small cell lung carcinoma, Endoplasmic reticulum aminopeptidase-1, Genetic association

## Abstract

An effective cytotoxic immune response to neoplastic cells requires efficient presentation of antigenic peptides to T lymphocytes by HLA class I (HLA-I) molecules. The HLA-I-bound peptide repertoire depends on antigen-processing machinery molecules. Aminopeptidase residing in endoplasmic reticulum 1 (ERAP1) trims peptides to the optimal length for HLA-I binding. Single nucleotide polymorphisms (SNPs) in the *ERAP1* gene result in changes in aminopeptidase activity and specificity. This may affect susceptibility to cancer. However, non-small cell lung carcinoma (NSCLC) has not been studied in this respect. We compared genotype and haplotype frequencies of four coding, nonsynonymous *ERAP1* SNPs, rs26653G > C, rs26618T > C, rs30187C > T, and rs27044C > G, in NSCLC occurring in two genetically distant populations, Chinese and Poles. We found associations of all four SNPs with NSCLC in Chinese but not in Poles. The differences in *ERAP1*-NSCLC associations might be explained by highly significant differences in SNP genotype frequencies between Chinese and Poles (except for rs26618). In accordance with this, the most frequent *ERAP1* haplotypes were distributed differently in cases versus controls in Chinese, but not in Poles. Our findings add to the differences between Orientals and Caucasians in genetics of disease susceptibility.

**Electronic supplementary material:**

The online version of this article (doi:10.1007/s00005-016-0436-4) contains supplementary material, which is available to authorized users.

## Introduction

Eradication of tumor cells is mediated by CD8^+^ cytotoxic T cells, which recognize peptide fragments of protein antigens presented to them by major histocompatibility complex class I molecules, human leukocyte antigen class I (HLA-I) in humans (Leone et al. 2013). Peptides bound and presented by HLA-I molecules are prepared by antigen-processing machinery composed of multiple components. Among these, immunoproteasome—a multiprotein complex in the cytosol—proteolytically cuts protein chains into peptides, which are transported to the endoplasmic reticulum by transporter associated with antigen processing molecules. Here, peptides are trimmed further by endoplasmic reticulum aminopeptidases 1 and 2 (ERAP1 and ERAP2) to fit better to the HLA-I peptide-binding groove (Fruci et al. 2014). The abnormalities of ERAP1 result in changes in HLA-I expression and impaired T cell responses. For example, several nonsynonymous single nucleotide polymorphisms (SNPs) located in the *ERAP1* gene, resulting in changes of the amino acid sequence, affect ERAP1 enzymatic properties (Reeves et al. 2013). The aim of the current study was to evaluate the associations of four coding and nonsynonymous SNPs (*rs26653G* > *C* [R127P], *rs26618T* > *C* [I276 M], *rs30187C* > *T* [K528R], *rs27044C* > *G* [Q730E]) in the *ERAP1* gene with NSCLC in two populations: Chinese Han and Polish Caucasians. The rationale behind comparison of these two populations was based on multiple differences described for Orientals and Caucasians in genetic associations with diseases (see “Discussion”).

## Materials and Methods

### Ethics Statement

All participants gave written and signed informed consent. The protocol was in accordance with the Helsinki Declaration and was approved by the Institutional Review Boards of the Institute of Medical Biology, Kunming (No. 2013[05]) and by the Bioethical Committee of Wrocław Medical University (No. 339/2010).

### Subjects

#### Chinese Patients and Controls

Case group included 420 patients who were diagnosed with non-small cell lung carcinoma (NSCLC) at the No. 1 and No. 3 Affiliated Hospitals of Kunming Medical University (China). The histological type of lung cancer was identified according to the World Health Organization (WHO 2004) classifications. Pathologic stages were determined according to the International System for Staging Lung Cancer (Groome et al. 2007). According to the histopathological reports, NSCLC included adenocarcinoma (AC), squamous cell carcinoma (SCC), and AC plus SCC. NSCLC patients with a history of primary cancer other than lung cancer were excluded from this study. Clinical characteristics and data, including sex, age, and histological type of cancer, are shown in Supplementary Table 1.

The healthy control group (Supplementary Table 1) included 385 subjects who had no family history of NSCLC and were recruited from an unselected population undergoing routine health checkups at the No. 1 and No. 3 Affiliated Hospitals of Kunming Medical University. All participants were self-reported to be ethnically Han and lived within roughly the same geographic region (Yunnan Province, China).

#### Polish Patients and Controls

A total of 317 patients with pathologically documented NSCLC (according to WHO criteria) were enrolled in our study by the Department of Pulmonology and Lung Cancer, Wrocław Medical University. Their clinical characteristics are shown in Supplementary Table 1 and described in more detail by Wiśniewski et al. (2015).

A total of 506 unrelated healthy Polish individuals, mostly from the same geographic region (Lower Silesia), were taken as a control group. After the Second World War the Polish population became highly homogeneously Polish by ethnicity (94.83%, Polish census of 2011).There was a weakly significant bias between Polish patients and controls in mean age (Supplementary Table 1).

### SNP Genotyping

Genomic DNA was extracted from peripheral leukocytes by a standard hydroxybenzene-chloroform method (China) or using an Invisorb Spin Blood Midi kit (Invitek, Berlin, Germany) following the manufacturer’s instructions (Poland). Four SNPs (*rs26653*, *rs26618*, *rs30187*, and *rs27044*) were genotyped. Each SNP genotyping was performed by the TaqMan assay (Applied Biosystems, Foster City, CA, USA). Some of the PCR products were characterized by direct sequencing (3100 Genetic Analyzer; Applied Biosystems, Tokyo, Japan).

### Statistical Analysis

The genotype and haplotype frequencies for these SNPs, Hardy–Weinberg equilibrium, linkage disequilibrium (LD) analysis and haplotype-association analysis were calculated using the SHEsis software (Li et al. 2009; Shi and He 2005); (http://analysis2.bio-x.cn/myAnalysis.php). The significance level for statistical tests was <0.05.

## Results

*ERAP1* SNP frequencies in patients versus controls were examined in Chinese and Polish populations. All SNPs were in Hardy–Weinberg equilibrium in all groups. We found highly significant differences between patients and controls in genotype frequencies for all four SNPs in Chinese but not in Poles (Table 1).

**Table 1:** Comparison of the distribution of four *ERAP1* single nucleotide polymorphisms (SNPs) between non-small cell lung cancer patients and healthy control individuals in Chinese and Poles

SNP	Group	Chinese *N* (%)				Poles *N* (%)			
		GG	GC	CC		GG	GC	CC	
rs26653	Patients	125 (29.8)	213 (50.7)	82 (19.5)	*χ* ^2^ = 16.87 *p* = 0.0002	172 (54.3)	120 (37.8)	25 (7.9)	*χ* ^2^ = 3.17 *p* = 0.2
[R127P]	Controls	76 (19.7)	194 (50.4)	115 (29.9)		258 (51.0)	219 (43.3)	29 (5.7)	
		TT	TC	CC		TT	TC	CC	
rs26618	Patients	189 (45.0)	195 (46.4)	36 (8.6)	*χ* ^2^ = 15.69 *p* = 0.0004	174 (54.9)	121 (38.2)	22 (6.9)	*χ* ^2^ = 0.07 *p* = 0.96
[I276 M]	Controls	227 (59.0)	134 (34.8)	24 (6.2)		281 (55.5)	192 (37.9)	33 (6.6)	
		CC	CT	TT		CC	CT	TT	
rs30187	Patients	137 (32.6)	203 (48.3)	80 (19.0)	*χ* ^2^ = 11.95 *p* = 0.0025	138 (43.6)	137 (43.2)	42 (13.2)	*χ* ^2^ = 1.07 *p* = 0.58
[K528R]	Controls	87 (22.6)	198 (51.4)	100 (26.0)		228 (45.0)	223 (44.1)	55 (10.9)	
		CC	CG	GG		CC	CG	GG	
rs27044	Patients	160 (38.1)	189 (45.0)	71 (16.9)	*χ* ^2^ = 17.47 *p* = 0.00016	170 (53.6)	118 (37.2)	29 (9.2)	*χ* ^2^ = 1.13 *p* = 0.57
[Q730E]	Controls	95 (24.7)	201 (52.2)	89 (23.1)		275 (54.3)	195 (38.6)	36 (7.1)	

Chinese: patients *N* = 420; controls *N* = 385. Poles: patients *N* = 317; controls *N* = 506

We also analyzed possible associations of *ERAP1* SNPs with different histopathological types of NSCLC (Supplementary Table 2). We observed differences in rs26653 and rs30187 genotype frequencies between squamous cell carcinoma and adenocarcinoma in Polish patients.

The significance was weak, because we had detailed clinical data for only 51.1% of Polish patients, thus reducing the size of the sample suitable for analysis to a half. Numbers of patients diagnosed with large cell carcinoma were too small to be analyzed.

No differences between squamous cell carcinoma and adenocarcinoma were found for the two other SNPs in Poles or for any *ERAP1* polymorphism in Chinese (Supplementary Table 2).

When we compared frequencies of tested polymorphisms between our control Chinese and Polish populations, we found highly significant differences for three SNPs (rs26653, rs27044, and rs30187); however, rs26618 had similar genotype frequencies in both populations (Table 2).

**Table 2:** Comparison of the distribution of four *ERAP1* single nucleotide polymorphisms (SNPs) between Chinese and Polish healthy populations

SNPs	Control groups	Genotypes *N* (%)			
		GG	GC	CC	
rs26653	Chinese	76 (19.7)	194 (50.4)	115 (29.9)	*χ* ^2^ = 138.16
[R127P]	Poles	258 (51.0)	219 (43.3)	29 (5.7)	*p* < 0.00001
		TT	TC	CC	
rs26618	Chinese	227 (59.0)	134 (34.8)	24 (6.2)	*χ* ^2^ = 1.07
[I276 M]	Poles	281 (55.5)	192 (37.9)	33 (6.6)	*p* = 0.58
		CC	CT	TT	
rs30187	Chinese	87 (22.6)	198 (51.4)	100 (26.0)	*χ* ^2^ = 62.38
[K528R]	Poles	228 (45.0)	223 (44.1)	55 (10.9)	*p* < 0.00001
		CC	CG	GG	
rs27044	Chinese	95 (24.7)	201 (52.2)	89 (23.1)	*χ* ^2^ = 95.46
[Q730E]	Poles	275 (54.3)	195 (38.6)	36 (7.1)	*p* < 0.00001

Chinese *N* = 385; Poles *N* = 506

**Fig. 1:**
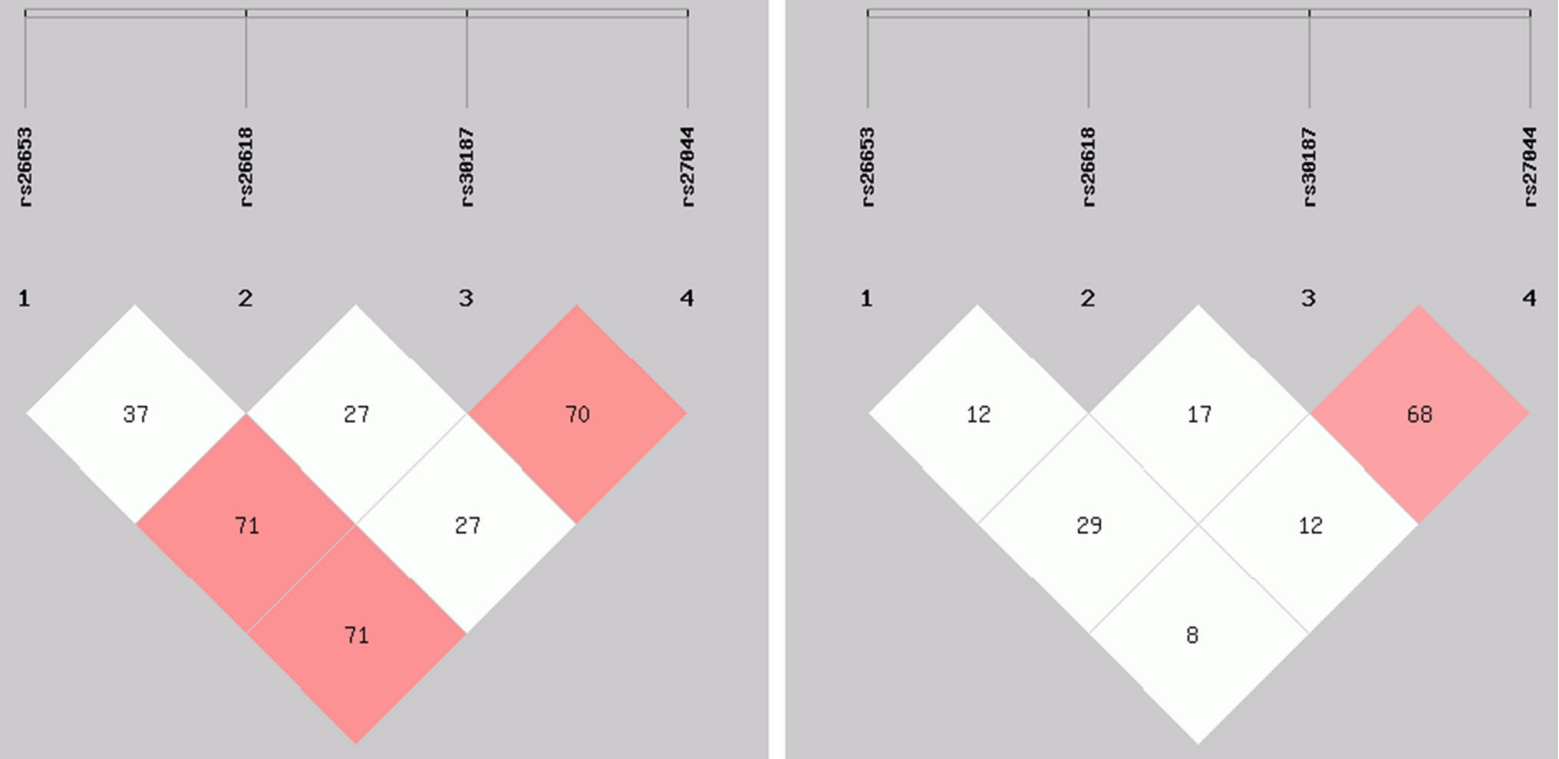
LD pattern of the four SNPs genotyped in the *ERAP1* gene in Chinese (*left*) and Poles (*right*). The plot shows *r* ^2^ × 100 value as a pairwise measure of LD. *Red* shading indicates strong LD

Chinese exhibited LD (*r* ^2^ 70–71) between rs26653 and rs30187, between rs26653 and rs27044, and between rs30187 and rs27044 (Fig. 1, left panel), whereas only two SNPs, rs30187, and rs27044, were in comparable LD (*r* ^2^ 68) in Poles (Fig. 1, right panel). rs26618, having similar genotype frequencies in both populations (Supplementary Table 2), was not in LD with any other SNP in Chinese and Poles (Fig. 1).

Combinations of individual SNPs in particular haplotypes were shown to generate a cumulative effect on peptide trimming and on the susceptibility to disease (Reeves et al. 2013). We see here that SNPs not associated individually with NSCLC in Poles were not related to the disease as haplotypes either, whereas the opposite is seen in Chinese (Supplementary Table 4): haplotype C1, consisting of NSCLC-protective alleles in all four positions, was strongly protective, but haplotype C2, possessing three SNPs predisposing to NSCLC, was associated with it. Haplotype C3, composed of three predisposing alleles (26653G, 30187C, 27044C) and one protective (26618T), was neutral, suggesting that one protective SNP balanced the other three. The remaining haplotypes were rare, but one of them, C6, containing three protective and one predisposing SNP, was nevertheless associated with disease. Haplotype frequencies in Chinese and Poles differed: the most frequent in Chinese, C1, was third (P3) in Poles, whereas P1, most frequent in Poles, was third (as C3) in Chinese (Supplementary Table 3).

## Discussion

We found here some associations of *ERAP1* SNPs (*rs26653G* > *C, rs26618T* > *C, rs30187C* > *T*, and *rs27044C* > *G*) with prevalence of NSCLC. Interestingly, the results for Chinese and Polish populations were different: genotypes seemingly protecting against NSCLC in Chinese were neutral in Poles. On the other hand, two SNPs apparently (albeit weakly) differentiated between squamous cell carcinoma and adenocarcinoma in Poles, but not in Chinese patients. All four SNPs tested here cause amino acid changes in the ERAP1 protein. Interestingly, it has been shown for rs26653 (R127P) to affect the level of ERAP1 protein expression (Mehta et al. 2009). Residues 127 and 528 are located in or close to the inter-domain region and presumably affect the conformational transition between the open (catalytically inactive but receptive to substrate binding) and closed (active but nonreceptive) states (Alvarez-Navarro and López de Castro 2014). Indeed, rs30187 (K528R) was found to decrease ERAP1 enzymatic activity (Goto et al. 2006). On the other hand, residue 730 is situated in the substrate-binding cavity; therefore, it potentially may affect peptide binding (Alvarez-Navarro and López de Castro 2014). In addition, each individual SNP has only a limited effect on ERAP1 activity (Reeves et al. 2013), and we also saw that it was difficult to predict the effect of a haplotype on the basis of its constituent SNPs (see “Results”).

The discrepancy between Chinese and Polish NSCLC patients versus controls may be partially due to different *ERAP1* genotype frequencies in these two populations: It can be seen in Supplementary Table 3 that homozygotes for minor alleles are, except for *rs26618*, highly significantly more frequent in Chinese than in Poles. No other populations have been tested so far for *ERAP1* polymorphisms in lung cancer. Therefore, we compared our results with those published earlier by Mehta et al. (2007) for human papilloma virus-induced cervical carcinoma in a Dutch population. Although two of the SNPs tested by us were associated with cervical carcinoma in Dutch (*rs26653* and *rs27044*), in contrast to our results with NSCLC in Poles, genotype frequencies in control Dutch and Polish groups were quite similar, in contrast to Chinese, with the exception of *rs26618*, which was distributed similarly in all three populations. Clear differences between Dutch and Chinese in distribution of *rs27044* and *rs26653* in patients versus controls were also seen: major alleles were less frequent in cases than in controls in Dutch, whereas the opposite was true for Chinese. For *rs26618*, Dutch cervical carcinoma patients were not different from controls, which was similar to what we observed in Polish, but not Chinese, NSCLC patients. Differences in genotype frequencies between Chinese and Poles were also reflected in strong LD between three out of four analyzed SNPs in the Chinese population, and between only two SNPs in the Polish population. This is confirmed by including in our comparison the data published for a Spanish population (Szczypiorska et al. 2011), which presented *r* ^2^ values strikingly similar to those of Poles but different from Chinese, except for rs30187–rs27044 LD, almost identical for all three populations (Supplementary Table 4).

Another possible explanation of the Chinese–Polish difference in *ERAP1* SNP associations with NSCLC, observed here, may lie in differences in *HLA* allele frequencies between Orientals and Caucasians (Gonzalez-Galarza et al. 2015). Some peptides presented by HLA class I molecules are closely dependent on ERAP1 trimming for optimal generation and may not be presented when a proper *ERAP1* allele is lacking. Other peptides are destroyed by some ERAP1 allotypes by over-trimming. Finally, some peptides are independent from ERAP1 because they fit the HLA molecule even without trimming (Fruci et al. 2014). It is conceivable, then, that different HLA alleles predominant in Chinese and Poles require distinct peptides for an immune response to NSCLC antigens, and peptides presented in Poles are insensitive to ERAP1, whereas those in Chinese require ERAP1-mediated trimming.

In conclusion, the following were observed: (1) great differences in frequencies of *ERAP1* SNPs between Chinese and Polish healthy populations; (2) four *ERAP1* polymorphisms that were found strongly associated with NSCLC in Chinese, but not in Poles; and (3) an association (albeit weak) of two *ERAP1* polymorphisms with distinct histopathological types of NSCLC in Poles, but not in Chinese.

Our results are novel, as no study on *ERAP1* polymorphism in lung cancer has been published so far. Therefore, these results should be confirmed using other populations of East Asians and Caucasians.

## Electronic supplementary material

Below is the link to the electronic supplementary material.

Supplementary material 1 (DOC 147 kb)
