# Supplementary material for: Single Nucleotide Polymorphisms of the ERAP1 Gene and Risk of NSCLC: A Comparison of Genetically Distant Populations, Chinese and Caucasian
Source: Arch Immunol Ther Exp (Warsz). 2017 Jan 12;64(Suppl 1):117–22. doi: 10.1007/s00005-016-0436-4 (PMC5334393; doi:10.1007/s00005-016-0436-4)
Supplement: Supplementary file 1 — Supplementary material 1 (DOC 147 kb) [file 5_2016_436_MOESM1_ESM.doc]

**Supplementary Table 1. Characteristics of Chinese and Polish non-small cell carcinoma (NSCLC) patients and controls**

|  | Chinese | | | Poles | | |
| --- | --- | --- | --- | --- | --- | --- |
|  | NSCLC | Controls | *p* | NSCLC | Controls | *p* |
| N | 420 | 385 |  | 317 | 506 |  |
| Ages ( years ) ± SD | 56.10±10.87 | 56.00±8.98 | 0.88 | 63.31±8.67 | 56.4±18.22 | 0.01 |
| Sex ( M/F ) | 288/132 | 260/125 | 0.76 | 232/85 | 339/167 | 0.06 |
| Histopathological type:  AC | 256 |  |  | 75 |  |  |
| SSC | 157 |  |  | 69 |  |  |
| AC+SSC | 7 |  |  | 0 |  |  |
| LCC | 0 |  |  | 17 |  |  |
| Undetermined | 0 |  |  | 156 |  |  |
| Clinical stage: |  |  |  |  |  |  |
| I | 67 (16.0%) |  |  | 63 (19.9%) |  |  |
| II | 68 (16.2%) |  |  | 35 (11.0%) |  |  |
| III | 149 (35.5%) |  |  | 95 (30.0%) |  |  |
| IV | 136 (32.3%) |  |  | 117 (36.9%) |  |  |

N: number; M/F: males/females; SCC: squamous cell carcinoma; AC: adenocarcinoma; LCC: large cell carcinoma.

**Supplementary Table 2. Comparison of the distribution of four *ERAP1* single nucleotide polymorphisms (SNPs) between squamous cell carcinoma and adenocarcinoma in Chinese and Poles**

| SNP | Group |  |  | Chinese  *N* (%) |  |  |  | Poles  *N* (%) |  |  |
| --- | --- | --- | --- | --- | --- | --- | --- | --- | --- | --- |
|  |  |  |  |  |  |  |  |  |  |  |
|  |  |  | **GG** | **GC** | **CC** |  | **GG** | **GC** | **CC** |  |
| rs26653 | SCC |  | 53 (26.1) | 114 (56.2) | 36 (17.7) | χ^2^=3.41 | 44 (63.8) | 17 (24.6) | 8 (11.6) | χ^2^=8.13 |
| [R127P] | AC |  | 43 (32.3) | 61 (45.9) | 29 (21.8) | *p*=0.18 | 36 (48.0) | 35 (46.7) | 4 (5.3) | *p*=0.017 |
|  |  |  |  |  |  |  |  |  |  |  |
|  |  |  | **TT** | **TC** | **CC** |  | **TT** | **TC** | **CC** |  |
| rs26618 | SCC |  | 89 (43.8) | 99 (48.8) | 15 (7.4) | χ^2^=1.45 | 37 (53.6) | 25 (36.2) | 7 (10.2) | χ^2^=1.23 |
| [I276M] | AC |  | 61 (45.9) | 58 (43.6) | 14 (10.5) | *p*=0.48 | 41 (54.7) | 30 (40.0) | 4 (5.3) | *p*=0.54 |
|  |  |  |  |  |  |  |  |  |  |  |
|  |  |  |  |  |  |  |  |  |  |  |
|  |  |  | **CC** | **CT** | **TT** |  | **CC** | **CT** | **TT** |  |
| rs30187 | SCC |  | 61 (30.0) | 106 (52.2) | 36 (17.7) | χ^2^=3.66 | 27 (39.1) | 28 (40.6) | 14 (20.3) | χ^2^=7.55 |
| [K528R] | AC |  | 45 (33.8) | 56 (42.1) | 32 (24.1) | *p*=0.16 | 32 (42.7) | 39 (52.0) | 4 (5.3) | *p*=0.02 |
|  |  |  |  |  |  |  |  |  |  |  |
|  |  |  |  |  |  |  |  |  |  |  |
|  |  |  | **CC** | **CG** | **GG** |  | **CC** | **CG** | **GG** |  |
| rs27044 | SCC |  | 33 (16.3) | 101 (49.8) | 69 (34.0) | χ^2^=3.05 | 33 (47.8) | 26 (37.7) | 10 (14.5) | χ^2^=4.86 |
| [Q730E] | AC |  | 27 (20.3) | 55 (41.4) | 55 (41.4) | *p*=0.21 | 39 (52.0) | 33 (44.0) | 3 (4.0) | *p*=0.08 |
|  |  |  |  |  |  |  |  |  |  |  |

SCC: squamous cell carcinoma; AC: Adenocarcinoma; *p* value for comparison SCC vs. AC

**Supplementary Table 3.** Estimated *ERAP1* haplotype frequencies for controls and NSCLC patients in Poles and Chinese

| Population | Haplotype ID | Haplotype sequence | Controls  % | Patients  % | OR (95%CI) | Haplotype-specific  *p*-value | Global  *p*-value |
| --- | --- | --- | --- | --- | --- | --- | --- |
| Poles | P1 | G-T-C-C | 34.7 | 31.7 | 0.865 (0.700–1.069) | 0.178 |  |
|  | P2 | G-C-C-C | 25.5 | 26.0 | 1.022 (0.814–1.282) | 0.853 |  |
|  | P3 | C-T-T-G | 14.0 | 12.3 | 0.856 (0.636–1.151) | 0.303 | 0.27 |
|  | P4 | G-T-T-G | 11.8 | 15.3 | 1.351 (1.012–1.803) | 0.041 |  |
|  | P5 | C-T-T-C | 7.2 | 7.3 | 1.009 (0.688–1.481) | 0.962 |  |
|  | P6 | C-T-C-C | 6.2 | 7.3 | 1.176 (0.793–1.744) | 0.418 |  |
|  |  |  |  |  |  |  |  |
| Chinese | C1 | C-T-T-G | 47.6 | 35.3 | 0.632 (0.517–0.774) | <0.00001 |  |
|  | C2 | G-C-C-C | 23.2 | 28.7 | 1.406 (1.122–1.762) | 0.003 |  |
|  | C3 | G-T-C-C | 20.2 | 21.5 | 1.135 (0.891–1.446) | 0.305 | <0.00001 |
|  | C4 | C-T-C-C | 4.3 | 2.5 | 0.585 (0.334–1.024) | 0.057 |  |
|  | C5 | C-T-T-C | 3.1 | 3.7 | 1.240 (0.722–2.129) | 0.43 |  |
|  | C6 | C-T-C-G | 0.1 | 3.3 | 58.56 (7.63–449.23) | <0.00001 |  |

SNPs in haplotypes listed in following order: rs26653, rs26618, rs30187, rs27044; Haplotypes with frequency <0.03 in both controls and patients has been not shown and not included in analysis. OR: odds ratio; 95%CI : 95%confidence interval

**Supplementary Table 4.** Comparison of linkage disequilibrium of tested *ERAP1* SNPs in Chinese, Poles and Spaniards

|  | rs26653 | rs26618 | rs30187 |
| --- | --- | --- | --- |
| rs26618 | 37 (C)  12 (P)  n.d. (S) |  |  |
| rs30187 | 71 (C)  29 (P)  26 (S) | 27 (C)  17 (P)  n.d. (S) |  |
| rs27044 | 71 (C)  8 (P)  6 (S) | 27 (C)  12 (P)  n.d. (S) | 70 (C)  68 (P)  67 (S) |

Numbers are r^2^ × 100 values as pairwise measure of linkage disequilibrium. 0 means that loci are in a complete linkage equilibrium. 100 means that loci are in a complete linkage disequilibrium.

C: Chinese; P: Poles (both from this report); S: Spaniards (Szczypiorska et al. 2011); n.d.: not done.
